# Anti-interferon armamentarium of human coronaviruses

**DOI:** 10.1007/s00018-025-05605-z

**Published:** 2025-03-13

**Authors:** Oyahida Khatun, Sumandeep Kaur, Shashank Tripathi

**Affiliations:** 1 Emerging Viral Pathogens Laboratory, Centre for Infectious Disease Research, Indian Institute of Science, Bengaluru, India; 2 Microbiology & Cell Biology Department, Biological Sciences Division, Indian Institute of Science, Bengaluru, India

**Keywords:** Innate immunity, Interferon, Interferon-stimulated genes, Human coronaviruses, Viral proteins, Immune dysregulation

## Abstract

Cellular innate immune pathways are formidable barriers against viral invasion, creating an environment unfavorable for virus replication. Interferons (IFNs) play a crucial role in driving and regulating these cell-intrinsic innate antiviral mechanisms through the action of interferon-stimulated genes (ISGs). The host IFN response obstructs viral replication at every stage, prompting viruses to evolve various strategies to counteract or evade this response. Understanding the interplay between viral proteins and cell-intrinsic IFN-mediated immune mechanisms is essential for developing antiviral and anti-inflammatory strategies. Human coronaviruses (HCoVs), including SARS-CoV-2, MERS-CoV, SARS-CoV, and seasonal coronaviruses, encode a range of proteins that, through shared and distinct mechanisms, inhibit IFN-mediated innate immune responses. Compounding the issue, a dysregulated early IFN response can lead to a hyper-inflammatory immune reaction later in the infection, resulting in severe disease. This review provides a brief overview of HCoV replication and a detailed account of its interaction with host cellular innate immune pathways regulated by IFN.

## Introduction

Coronaviruses are a large group of viruses that belong to the order Nidovirale, family Coronaviridae, and suborder Coronavirineae. This family consists of four genera: *alphacoronavirus*, *betacoronavirus*, *gammacoronavirus,* and *deltacoronavirus*. Human coronaviruses belong to the *alpha* and *betacoronavirus genera* and cause respiratory disease; however, *gamma* and *deltacoronaviruses* have a broader host range encompassing mammalian and avian species [1, 2]. Before COVID-19 pandemic, 6 human coronaviruses (HCoVs) were reported. While Avian and Murine coronaviruses were discovered earlier, the first HCoV were isolated in the mid-1960s, named HCoV-OC43 and HCoV-229E, belonging to *alpha* and *betacoronaviruses* respectively [1, 3]. HCoV-OC43 and HCoV-229E, with other two human coronaviruses, HCoV-NL63 (*alphacoronavirus*) and HCoV-HKU1 (*betacoronavirus*), have been prevalent in the human population, and known to induce seasonal and mild respiratory diseases, associated with ‘common cold’ like symptoms [1]. However, two other important viruses of this family, severe acute respiratory syndrome coronavirus (SARS-CoV) and Middle East respiratory syndrome coronavirus (MERS-CoV), are highly pathogenic [4]. SARS-CoV emerged in November 2002 in Guangdong province, China. By June 2003, it had affected ~ 8000 people in 25 countries across five continents, and 774 people lost their lives [5]. MERS-CoV emerged in 2012 in Saudi Arabia and led to an outbreak spanning 27 countries, affecting 2,574 people and 885 deaths in 3 years [6] (https://www.emro.who.int/health-topics/mers-cov/mers-outbreaks.html). SARS-CoV-2, the etiological agent of the pandemic COVID-19, was the latest addition to *betacoronavirus* genera. COVID-19 first emerged in Wuhan, China, in December 2019 and then turned into the biggest global public health crisis of the current century [7, 8]. By February 2025, more than 700 million COVID-19 infections and 7 million deaths had been reported globally [https://www.worldometers.info/coronavirus/].

The cellular innate immune response driven by interferons (IFNs), the cornerstone of the antiviral defense mechanisms, presents a significant barrier that viruses must surmount upon entering the host [9]. Thus, viruses encode multiple proteins that utilize a plethora of strategies to antagonize the IFN induction and subsequent immune response [10]. Coronavirueses are no exception [11, 12]. Here in this review, we provide a detailed account of evasion and antagonism mechanisms that exist in the anti-IFN armamentarium of human coronaviruses and discuss their implications for viral pathogenesis and antiviral strategies.

## IFN-dependent cellular antiviral response

The innate immune pathway is the front line of the host safeguarding mechanism that starts with the detection of pathogen-associated molecular patterns (PAMP) by cellular sensors and pattern recognition receptors (PRRs) [13]. The most widely studied nucleic acid sensors that elicit IFN productions are RIG-I-like receptors (RLRs), cyclic GMP–AMP synthase (cGAS), Toll-like receptors (TLRs), NOD-like receptors (NLRs), and AIM-2 like receptors (ALRs) [14–16]. RLRs are cytosolic RNA sensors [17]. Currently identified members of RLRs are RIG-I, MDA5, and LGP2 [17, 18]. Upon viral RNA encounter, stable RLR-RNA interaction displaces the CARD domain, allows oligomerization of RIG-I and MDA5 [18, 19], and interacts with MAVS [20–23]. Activated MAVS interacts with adapter protein TRAF3, activating two important kinases, TBK1 and IKKε. These kinases, in turn, phosphorylate and activate transcription factors interferon regulatory factors 3 and 7 (IRF3 and IRF7). Activated IRFs homo- and hetero dimerize, translocate to the nucleus, and drive the transcription of type I IFNs by binding to the positive regulatory domains (PRD) in the promoter region of the IFN gene [24]. The complete induction of IFNs needs the assembly of IRF dimers on PRDI and PRDIII cis-regulatory elements along with NFkB and AP1 transcription factors to form the complete enhanceosome [25]. Another important PRR is cytosolic DNA sensor cGAS. Upon DNA binding, monomeric cGAS dimerizes to form a secondary messenger molecule- 2’-3’ cyclic GMP-AMP (cGAMP), that binds and activates the STING (Stimulator of Interferon Genes) [26, 27]. The encounter of activated STING with TBK1 results in its phosphorylation, which in turn recruits IRF3. Besides the obvious role in IFN induction, STING drives other functions like inflammation and autoimmunity via IKKε, NFkB [28]. The collateral damage upon a virus infection can lead to disruption of mitochondrial integrity and leakage of DNA into the cytosol, where its recognition by cGAS can activate the IFN system [29]. The TLR family is a relatively ancient family of innate immunity proteins, and to date, 10 TLRs have been identified in humans. Among all TLRs, nucleic acid sensors are TLR3 (dsRNA), TLR7 and 8 (ssRNA), and TLR9 (CpG-DNA) [30]. TLR activation upon PAMP recognition translates to IFN response through the NFkB via TRAF6 activation or IRF3 via TRAF3 [31].

IFNs are secretory molecules and act in an autocrine and paracrine manner where they bind with the IFN alpha and beta receptor (IFNAR) and activate the Janus kinase 1 (JAK1) and tyrosine kinase 2 (Tyk2). These kinases phosphorylate transcription factors called STATs (signal transducer and activator of transcription), particularly STAT1 and STAT2 [32, 33]. Phosphorylated STATs bind with IRF9 and form a complex called interferon stimulatory gene factor 3 (ISGF3) and translocate to the nucleus where it binds to interferon response element (ISRE) in interferon-stimulated gene (ISG) promoter. These induce the transcription of a plethora of ISGs [33, 34]. RLR pathway activation is very tightly regulated by several post-translational modifications (PTMs). TRIM25, an E3 ligase, adds K63-linked polyubiquitination at the K172 residue in the CARD domain of RIG-I [35]. This modification stabilizes the RIG-I, which is required for MAVS binding and downstream signal activation [36]. Overall, IFN induction and downstream signaling are critical for mounting localized innate immune responses to viral infections and recruiting the immune effector cells at the site of infection to initiate adaptive immune responses.

## HCoV infection and replication

The coronavirus genome is a non-segmented, positive sense, single-stranded RNA of around 27–30 Kb, which is translation-ready. The 5′ end of the genome contains open reading frames ORF1a and ORF1b that encode polyproteins pp1a, and pp1ab, respectively. The latter is produced by -1 ribosomal frameshift at the short overlap of ORF1a and 1b, producing 16 nonstructural proteins (NSPs) [37]. The 3′ end encodes for 4 structural proteins and several accessory proteins. Structural proteins are spike (S), envelope (E), membrane (M), nucleocapsid (N), and several accessory proteins which vary in numbers among the HCoVs [38]. The primary function of the NSPs is replicase complex formation, whereas structural proteins are involved in virion formation. The accessory proteins are the key modulators of the host cellular immune pathways [39].

**Fig. 1:**
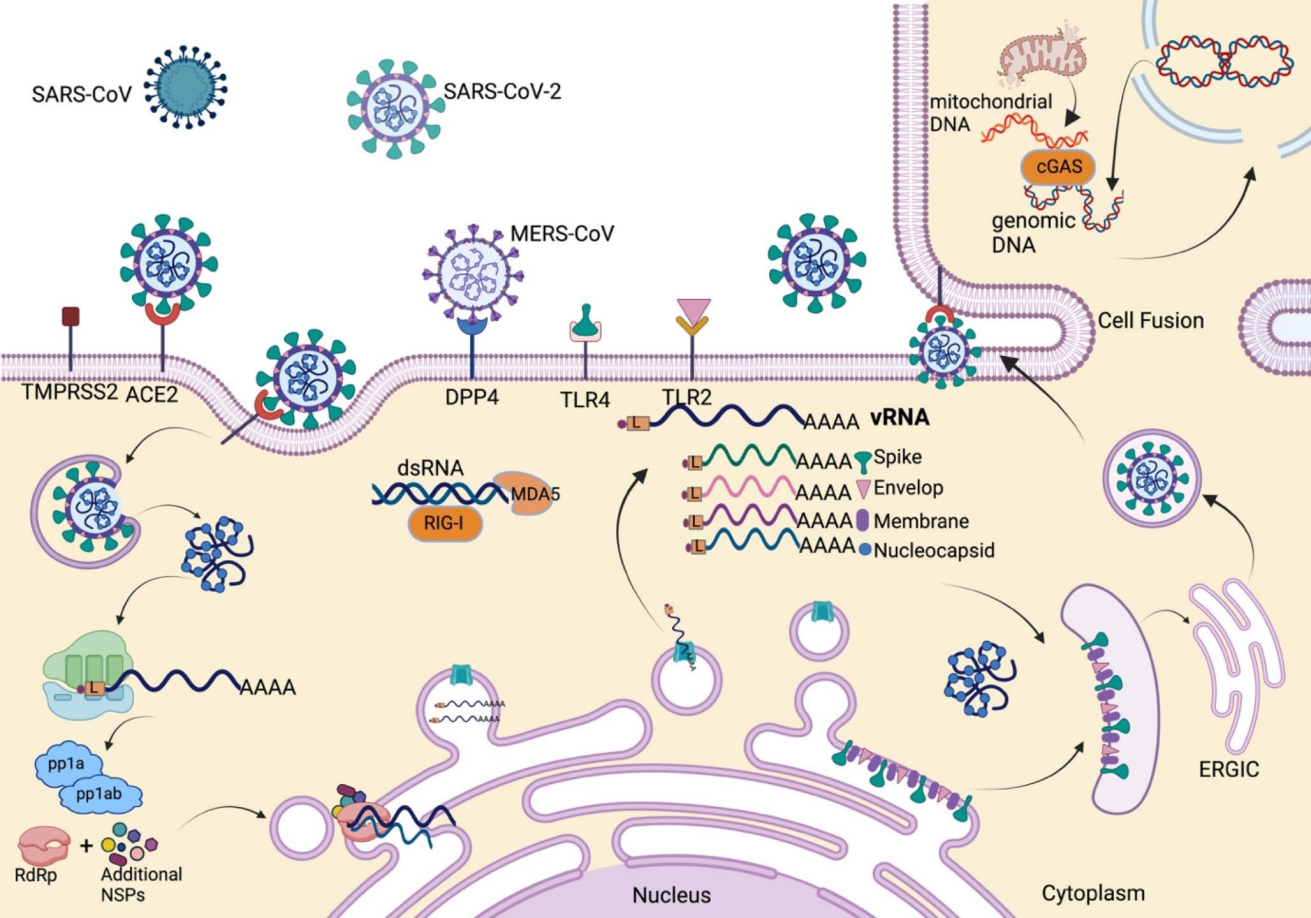
HCoV replication and interferon-mediated cellular innate immunity. Upon binding with a receptor on the cell surface, the coronavirus virus enters via endocytosis. Following entry, the genome is uncoated and released in the cytoplasm, which undergoes immediate translation and produces pp1a and pp1ab. After polyprotein processing, RdRp, along with NSPs, produces RTC and initiates replication and transcription inside DMVs. These RNAs are later exported to the cytoplasm through membrane pores (shown in green on the DMV membrane) and produce structural and accessory proteins. They, in turn, translocate to ER with N-coated vRNA and migrate to ERGIC. Then, mature virion particles are released in the extracellular region via exocytosis. While the virus searches for the receptor, S and E proteins are recognized by TLR4 and TLR2, respectively, followed by activation of the TLR pathway. Similarly, during replication, the dsRNA intermediate is recognized by RIG-I and MDA-5 and activates the RLR pathway. S expressed on the infected cell surface is often recognized by the ACE2 receptor of neighboring cells and leads to cell fusion. That, in turn, results in nuclear membrane rupture and release of genomic DNA in the cytoplasm, which activates the cGAS-STING pathway. Parallelly, mitochondrial damage due to virus infection leads to the release of mitochondrial DNA in the cytoplasm, activating cGAS

Human coronaviruses have respiratory tropism, although they can infect a variety of different cell lines of various tissue origins. Angiotensin-converting enzyme 2 (ACE2) acts as a receptor for SARS-CoV, HCoV-NL63, and SARS-CoV-2 entry [40–42]. Conversely, MERS-CoV utilizes dipeptidyl peptidase or DPP4 (also known as CD26) as a functional receptor for entry [43]. HCoV-229E uses aminopeptidase N (APN), while HCoV-OC43 and HCoV-HKU1 use 9-O-acetylated sialic acid as a receptor [41]. SARS-CoV and SARS-CoV-2 entry have been extensively studied, where spike protein plays a primary role in virus entry and initiates the process by binding with ACE2. Spike protein is composed of two subunits, S1 in the N-terminus and S2 in the C-terminal domain for receptor binding and fusion, respectively [44]. Upon receptor-mediated endocytosis, host protease Cathepsin L cleaves between the S1-S2 subunit to separate the RBD-fusion domain. Subsequent cleavage occurs inside S2 (S2′), unveils the peptide responsible for fusion, and allows the insertion of this peptide into the host membrane. Heptad repeats inside S2 combine to construct a bundle of six-helix which in turn allows the amalgamation of the host-virus membrane and results in fusion [45]. SARS-CoV-2 entry is pre-activated by host protease pro-protein convertase furin, which cleaves the polybasic site at the S1-S2 margins during virus maturation and enhances the entry and infectivity [46]. Transmembrane protease/serine subfamily 2 or TMPRSS2 can also activate S by promoting S2′ cleavage and inducing virus-cell membrane fusion, even in the presence of a cathepsin inhibitor [47]. Endosomal acidification leads to the uncoating of the genome and its release into the cytosol, followed by the translation and production of pp1a and pp1ab. These polyproteins are processed by the NSP3-encoded papain-like protease (PLpro), NSP5-encoded serine-type protease, main protease or chymotrypsin-like protease (Mpro or 3Clpro), and produce 16 nonstructural proteins (NSP1-16). NSP2-11 acts as a cofactor for replication-transcription and participates in inducing double-membrane vesicle (DMV) formation. NSP12-16 forms the replication-transcription complex (RTC), where NSP12 is the RNA-dependent RNA polymerase (RdRp) [4]. In coronaviruses, replication-transcription takes place inside DMVs [48–50]. Viral replication initiates by synthesizing a full-length negative sense RNA, which acts as a template for genomic vRNA synthesis. The discontinuous transcription process produces a nested set of 5′ and 3′ co-terminal sub-genomic RNAs (sgRNA), which act as a template for making positive-sense RNAs. These RNAs are released from DMVs to the cytosol and translated into structural and accessory proteins [51]. Next, structural proteins translocate to and assemble in the ER-Golgi intermediate compartment (ERGIC). M protein interacts with the C-terminus of N, which is bound with virion RNA, thus enhancing virion maturation. Then, mature virions bud out from infected cells via exocytosis [52]. These shared steps of human coronavirus infection and replication are depicted in Fig. 1.

## HCoV sensing by cellular PRRs

During viral entry, SARS-CoV-2 structural protein spike is recognized by cell surface PRR TLR4, whereas TLR2 recognizes envelope and activates downstream signaling [53, 54]. During replication and transcription, dsRNA intermediates of coronaviruses are recognized by RLRs like RIG-I and MDA-5 [55]. The role of RIG-I in governing vRNA detection remains elusive. Although some studies showed that different regions of vRNAs, including 3′ UTR, can be sensed by RIG-I [56, 57], later studies claimed that RIG-I is not the primary sensor of SARS-CoV-2 detection. Instead, MDA-5 plays a central role in recognizing SARS-CoV-2 RNA. Multiple studies have reported MDA-5 as the primary sensor and essential for type I IFN pathway induction [58–60]. Some studies also show that LGP2 is another sensor [60]. Among the human coronaviruses, RIG-I acts as a sensor for MERS-CoV, whereas MDA5 is a sensor for MERS-CoV and HCoV-229E [61, 62]. Multiple TLRs also participate in sensing viral cues, e.g., TLR2 and 3 (SARS-CoV) and TLR7 (SARS-CoV and MERS-CoV) [63–66]. At a later stage of infection, SARS-CoV-2 infected cells express S at the cell surface that often binds with the ACE2 receptor present at the neighboring cell surface and results in cell-cell fusion or syncytia formation [67]. Cell fusion leads to nuclear rupture, micronuclei formation, and thus the cytoplasmic release of genomic DNA, which triggers the cGAS-STING signaling axis [68, 69]. Loss of mitochondrial homeostasis during virus infection also results in damage and leakage of mitochondrial DNA. That, in turn, activates the cGAS-STING pathway and results in discerning activation of the NFkB pathway and subsequent proinflammatory cytokine production [70, 71]. For a more detailed review of cellular innate immune pathways, the role of interferons [24, 72, 73], and an overview of the SARS-CoV-2 genome, proteome, and replication cycle, readers may refer to the cited references [4].

## HCoV strategies of IFN-evasion and antagonism

**Fig. 2:**
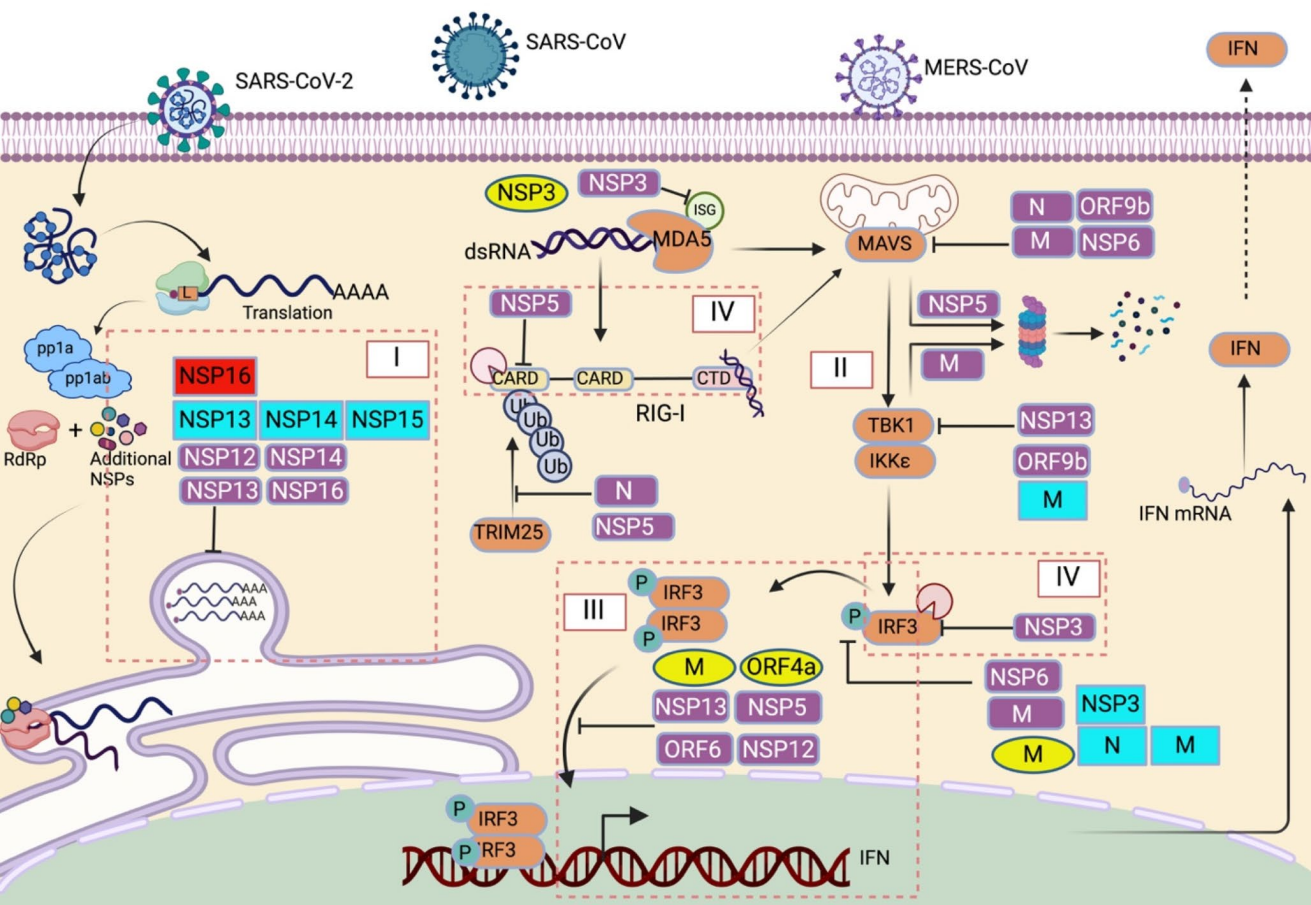
Evasion of RLRs and Inhibition of IFN induction by HCoV proteins. Replication intermediates generated during infection are recognized by RIG-I/MDA-5. Activated RIG-I/MDA-5 interacts and activates MAVS and initiates a signaling cascade that results in the activation of IKKε/TBK1. This kinase phosphorylates IRF3 transcription factors that, in turn, activate the transcription of IFN. Here, different strategies of IFN induction inhibition are mentioned inside the box, where strategy I is evasion from RLR-sensing, II is inhibition of RLR sensing and activation by HCoV proteins, III is targeting of IRFs to prevent IFN induction, and IV is proteolytic targeting of antiviral proteins. Coronavirus proteins of SARS-CoV-2 (shown in purple color), MERS-CoV (yellow color), SARS-CoV (turquoise), and HCoV-229E (red) adopt different strategies to inhibit the IFN induction. Some common mechanisms of viral proteins are cleaving the host proteins, inhibiting phosphorylation, targeting for proteasomal degradation, and inhibiting nuclear translocation of transcription factors

A large number of HCoV proteins, especially the accessory and nonstructural proteins, modulate cellular innate immunity. While most of them act negatively on IFN-mediated antiviral response, spike protein in a specific context can activate the inflammatory immune response. Several different strategies are utilized by HCoV proteins for these purposes (Fig. 2), which are divided into seven categories.

### Evasion from RLR-sensing

Cytosolic sensors are always alert and survey the cytosol to detect foreign features of RNA. After entry and genomic RNA release in the cytoplasm, the N protein dissociates from the genome, allowing immediate translation by host translational machinery [74]. To shield the replication intermediate, dsRNA, from cytosolic PRRs, HCoVs replicate inside DMVs [48–50]. In SARS-CoV-2, SARS-CoV, and MERS-CoV, the nonstructural proteins NSP3, NSP4, and NSP6 participate in inducing DMV formation to compartmentalize the replication [75–77]. Furthermore, SARS-CoV and SARS-CoV-2 modify their 5′-PPP structure to mimic the host mRNA and avoid detection by sensors. Four coronavirus enzymes work together to execute this (Fig. 2) [78, 79]. First, NSP13, which encodes for helicase, also has RNA triphosphatase activity, hydrolyzes the 5′ γ-phosphate from the 5′-ppp, and generates 5′-ppN [80, 81]. Second, a guanylyltransferase transfers a guanine monophosphate and generates GpppN-RNA. Third, NSP14, which encodes for an N7-methyltransferase (N7-MTase), methylates the guanine at the N7 position and generates ^m7^GpppN-RNA [82, 83]. Finally, NSP16, which encodes for a (SAM)-dependent-2′-O-methyltransferase, adds a methyl group to the ribose at the 2′-O position and creates the cap structure ^m7^GpppNm-RNA. NSP10 acts as a cofactor in this step [78, 84–87]. NSP16-mediated methylation for the cap structure formation is also reported for HCoV-229E [62]. Even though mRNA capping is not shown for MERS-CoV, the structural similarity of the NSP10-16 complex between SARS-CoV and MERS-CoV suggests the possibility of MERS-CoV having a similar mechanism to avoid host detection of viral RNA [88]. Since the viral mRNA has a cap structure, it can camouflage and proceed with the translation. However, during replication and transcription, dsRNA formation is inevitable. Thus, the virus minimizes the accumulation of negative-sense RNA and also keeps them away from cytosolic sensors. NSP15 of SARS-CoV-2 possesses uridine-specific endonuclease activity (NendoU) that cleaves the 5′-polyuridin sequence from negative-sense viral RNA and prevents its accumulation (Fig. 2) [89, 90].

### Inhibition of RLR sensing and activation by HCoV proteins

Even after capping the mRNA and compartmentalizing the replication, it is improbable to shut off the innate immune pathway completely. Hence, the virus utilizes different proteins to interfere with the IFN induction pathway at multiple steps as additional measures (Fig. 2). SARS-CoV-2 N protein inhibits IFNβ promoter activation by targeting the very first protein of the immune pathway. It directly interacts with RIG-I and TRIM25 and impairs the K63-linked polyubiquitination to prevent RIG-I activation [91, 92]. NSP5 also prevents the interaction between RIG-I and TRIM25, thus impairing RIG-I activation [93]. SARS-CoV-2 ORF6, on the other hand, targets TRIM25 for proteasomal degradation, thus diminishing the K63-ubiquitinated RIG-I [94]. MERS-CoV PLpro prevents RIG-I, MDA5, and MAVS-mediated IFN promoter activation and reduces the IFN mRNA level [95]. SARS-CoV-2 NSP7 blunts the IFN response by blocking the establishment of the RIG-I/MDA5-MAVS signalosome complex [96]. On the other hand, SARS-CoV-2 NSP5 targets MAVS for proteasomal degradation by promoting its K48-linked ubiquitination [97]. Similarly, M and N proteins are shown to impair MAVS aggregation formation and recruitment of downstream signaling components [98, 99]. ORF10 translocates to mitochondria, interacts with mitophagy receptor Nip3-like protein X (NiX) and LC3B, and targets MAVS for mitophagy-mediated degradation [100]. Other proteins of SARS-CoV-2 that target MAVS signaling are NSP6 and ORF9b, where ORF9b interacts with TOM70 [101, 102]. SARS-CoV ORF3b antagonizes RIG-I and MAVS-mediated IFNβ activation, where it localizes to the mitochondrial outer membrane [103]. TBK1/ IKKε are two pivotal kinases in the IFN induction pathway. SARS-CoV M protein interacts with RIG-I, TBK1, IKKε, and TRAF3 and prevents the formation of the TRAF3·TANK·TBK1/IKKε complex and thereby inhibits IRF3/IRF7 activation [104]. MERS-CoV M protein interacts with adapter protein TRAF3 and disrupts TRAF3-TBK1 association, which hinders IRF3 phosphorylation. The N-terminal transmembrane domain is crucial for this antagonistic activity [105]. MERS-CoV ORF4b, on the other hand, directly interacts with TBK1-IKKε and prevents their interaction with MAVS, thus inhibiting IRF3 phosphorylation and downstream induction pathway [106]. Multiple SARS-CoV-2 proteins like NSP6, NSP13, and ORF9b interact directly with TBK1, where NSP13 and ORF9b is shown to inhibit TBK1 phosphorylation [107, 108]. M protein, on the other hand, promotes TBK1 for degradation by K48-linked ubiquitination and suppresses the downstream signaling pathway [109].

### Targeting of IRFs to prevent IFN induction

Multiple coronavirus proteins are shown to target IRF3 activation. SARS-CoV ORF3b, ORF6, and N protein inhibits IRF3 activation [110]. SARS-CoV NSP7 and NSP15 are also demonstrated as interferon antagonists; however, the detailed mechanism is not yet elucidated [111]. MERS-CoV ORF4a, ORF4b, ORF5, and M protein inhibit nuclear translocation of IRF3 upon SeV infection [112]. MERS-CoV ORF4b competitively binds to the nuclear import adapter IMPα3, thus inhibiting the NFkB-dependent innate immune pathway [113]. SARS-CoV-2 ORF3b has yet to be studied in detail; however, it is an interferon antagonist [114]. SARS-CoV-2 M protein hampers the phosphorylation, nuclear relocation, and hence activation of IRF3 [115]. Another study demonstrated that the M protein inhibits the interaction of IRF3 with importin karyopherin subunit alpha-6 (KPNA6) and elucidated the mechanism of inhibition of nuclear translocation [116]. NSP5, NSP12, NSP13, and ORF6 of SARS-CoV-2 also inhibit the IRF3 nuclear translocation [117–120]. However, expression of HCoV-HKU1 M protein does not affect IFN production [121] (Fig. 2). The transmembrane domain of SARS-CoV PLpro (PLpro-TM) physically interacts with TRAF3, TBK1, IKKε, STING, and IRF3, and disrupts the interaction between the components in STING-TRAF3-TBK1 complex. Using its deubiquitinase activity, PLpro also reduces the ubiquitinated levels of RIG-I, STING, TRAF3, TBK1, and IRF3 in the STING-TRAF3-TBK1 complex. These collectively result in reduced IRF3 phosphorylation and dimerization [122]. HCoV-NL63 is shown to block STING dimerization and negatively regulate the assembly of STING-MAVS-TBK1/IKKε, which affects IRF3 activation [123]. SARS-CoV-2 NSP5 protease, on the other hand, prevents the K63-activating ubiquitination of STING protein [124]. In SARS-CoV-2, NSP6 activates PERK-eIF2A-mediated autophagy, leading to autophagy-mediated STING degradation [125]. Furthermore, the ORF3a protein of SARS-CoV-2 has been implicated in inhibiting STING-mediated autophagic flux, but its IFN inhibition has not been reported [126]. Additionally, another study reported that the ORF10 protein inhibits STING-TBK1 interaction and STING oligomerization [127].

### Inhibition of JAK-STAT signaling

**Fig. 3:**
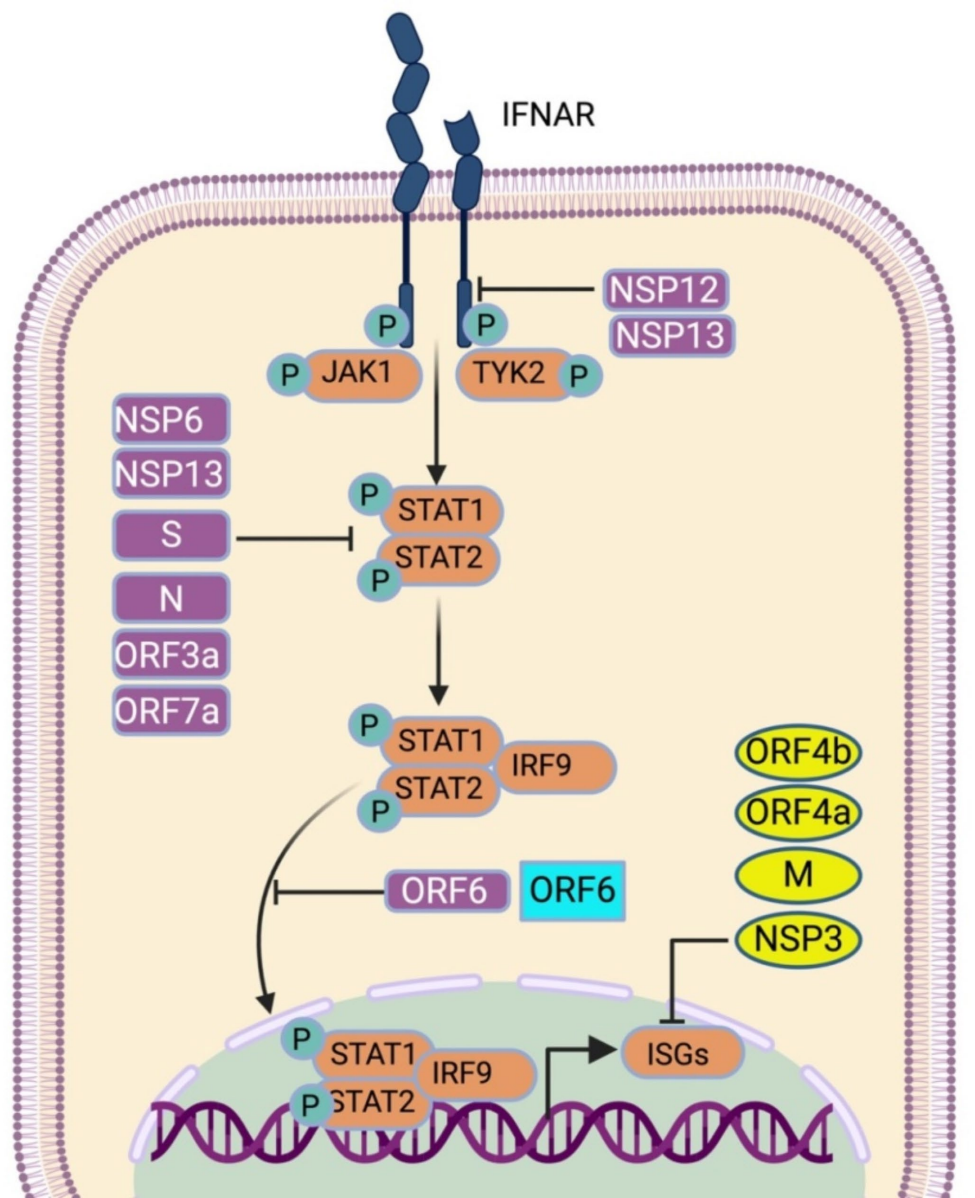
Inhibition of IFN signaling by HCoV proteins. Upon secretion in the extracellular milieu, IFN binds to the receptor IFNAR, induces the dimerization of the receptor, and brings the receptor-associated kinase JAK1 and TYK2 in close proximity. As a result, they transphosphorylate each other and promote activation and phosphorylation of STAT1 and STAT2. These, in turn, bind with IRF9 and translocate to the nucleus, where it activates ISG transcription. HCoV proteins interfere with every step of this innate immune pathway. Here, SARS-CoV-2 proteins are shown in purple, MERS-CoV proteins in yellow, SARS-CoV proteins in turquoise, and host proteins in orange.

To ablate the antiviral state completely, HCoVs efficiently block the IFN signaling downstream of IFN induction (Fig. 3). IFN binds to the IFNAR and activates the JAK-STAT pathway to produce ISGs. The NSP13 and NSP14 of SARS-CoV-2 reduced the endogenous level of IFNAR1. SARS-CoV-2 NSP14 targets the IFNAR1 for lysosomal degradation as Bafilomycin A1 treatment rescues the protein level. NSP14 is also shown to inhibit STAT1 phosphorylation [128]. NSP6 is shown to inhibit the STAT1 (Y701) and STAT2 (Y689) phosphorylation and subsequent IFN signaling [108]. NSP13 also interacts with STAT1 and prevents JAK1 from phosphorylating STAT1 [129]. Among the structural proteins of SARS-CoV-2, spike protein directly interacts with STAT1 with its S1 subunit and suppresses phosphorylation and nuclear translocation [116]. In addition, N protein competitively binds to STAT1/STAT2 and interferes with their interaction with JAK1/TYK2. It also reduces the phosphorylation of STAT1 and STAT2 and also suppresses their nuclear translocation [130]. N-terminal 1-361 amino acids are sufficient for this IFN antagonism [130]. ORF3a protein upregulates SOCS1, an E3 ligase that negatively regulates IFN signaling, which dampens STAT1 phosphorylation and accelerates JAK2 degradation in a ubiquitin-proteasome-dependent manner [131].

Nuclear translocation inhibition of STAT transcription factors by ORF6 has been studied quite well by multiple researchers. Nuclear envelopes allow the macromolecules to shuttle between cytoplasm and nucleus via the gatekeeper, nuclear pore complex or NPC, which coalesces the outer and inner nuclear membrane and forms an aqueous channel. NPC is comprised of different proteins called nucleoporins (Nups), which interact with karyopherin family proteins. This karyopherin consists of importins and exportins that recognize nuclear localization signal (NLS) and nuclear export signal (NES) [132]. SARS-CoV-2 ORF6 translocates to the NPC and interacts with nucleoporin 98 (Nup98) and ribonucleic acid export factor 1 (Rae1) to impede the docking of karyopherin/importin complex and disrupt nuclear translocation (Fig. 3) [133]. Methionine residue at position 58 of SARS-CoV-2 ORF6 is crucial for this activity, as the M58R mutation disrupts the interaction with the Nup98-Rae1 complex and abrogates the IFN antagonism [133]. Previous studies on SARS-CoV ORF6 show that ORF6 binds to karyopherin-α2 and tethers karyopherin-β1 on internal membranes, disrupting the complex formation associated with STAT1 nuclear import [134]. SARS-CoV-2 ORF7a inhibits the STAT2 phosphorylation but not STAT1 [135]. MERS-CoV PLpro inhibits the production of proinflammatory cytokines like CCL5 and CXCL10 [95]. MERS-CoV ORF4a, ORF4b, and M protein reduce ISG54 and ISG56 mRNA levels, thus ISRE promoter activation; however, ORF5 has minimal effect on ISRE promoter activation [112]. Ectopically expressed SARS-CoV-2 ORF8 results in aggregate formation in the cytosol and the nucleus and inhibits basal expression of ISGs, i.e., DHX58, ZBP1, MX1, and MX2. N-terminal amino acids 1–18 SARS CoV2 ORF8 possess intrinsic aggregation characteristics [136].

### Blockage of mRNA nuclear export in the host cell

**Fig. 4:**
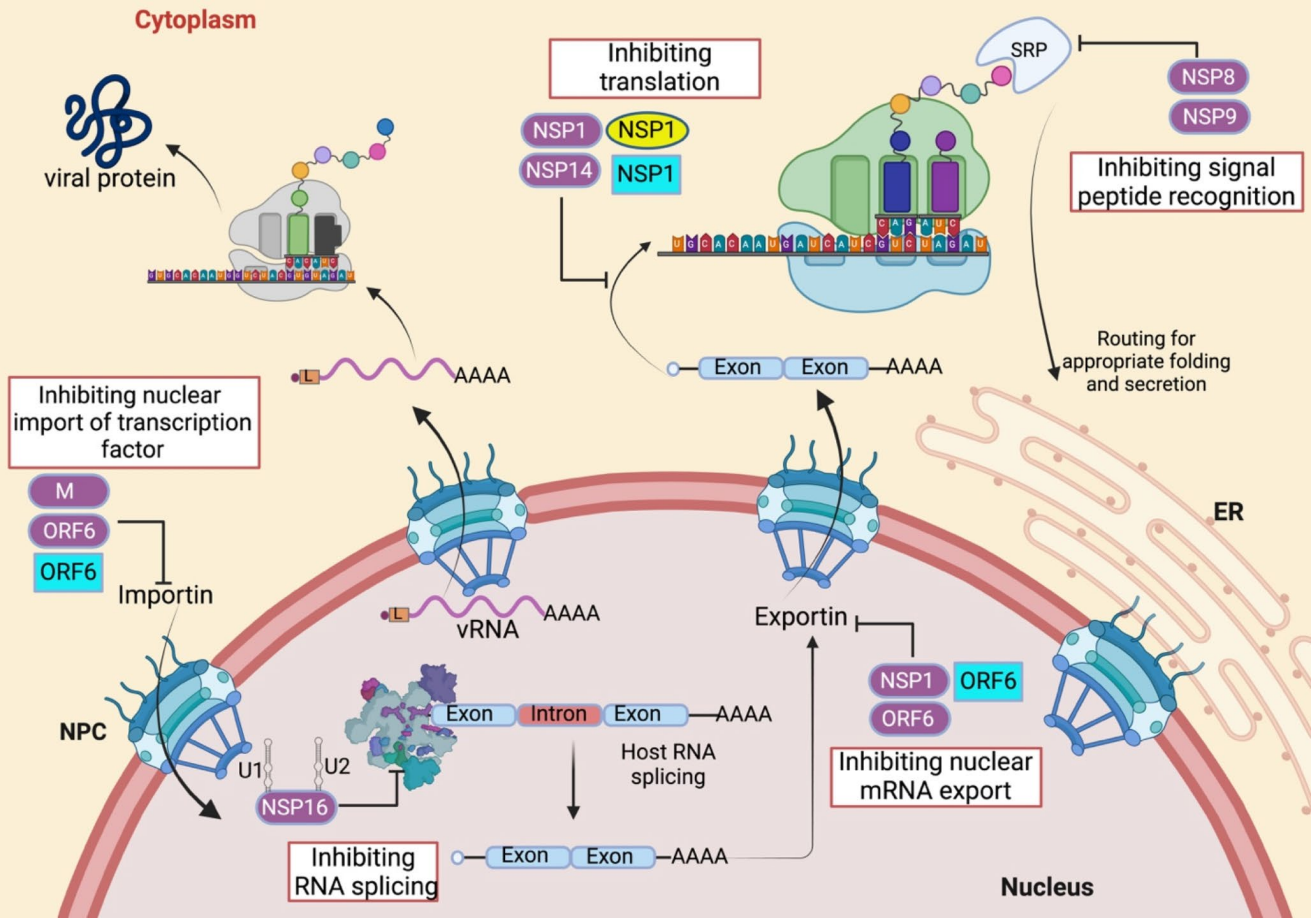
HCoV interference with host mRNA transport and translation. The bidirectional nucleo-cytoplasmic shuttle system is essential for the host immune response. Upon activation, transcription factors translocate to the nucleus with the help of importin, bind to the promoter of antiviral genes, and activate transcription. These transcripts are spliced to form mature mRNA with the aid of spliceosome and exported to the cytoplasm through NPC after association with receptors exportin. In the cytoplasm, mRNAs are translated by host ribosome, and based on recognition of signal peptide by SRP, they translocate to ER for proper folding and secretion and membrane insertion. Multiple SARS-CoV-2 (purple color), MERS-CoV (yellow color), and SARS-CoV (turquoise color) proteins interact with host proteins and inhibit nuclear translocation of proteins, mRNA export from the nucleus, RNA splicing, host proteins translation, and proper folding. However, mRNAs with viral leader sequences are selectively translated and produce viral proteins

Viruses often adapt elegant approaches to undertake the host nucleo-cytoplasmic shuttle system to elude the immune pathways. One of the mechanisms is to restrain the nuclear export of host mRNAs [132]. Multiple SARS-CoV-2 proteins are shown to target host mRNA export (Fig. 4). Apart from inhibiting STAT1/2 nuclear translocation, both SARS-CoV and SARS-CoV-2 ORF6-Nup98-Rae1 interaction is also responsible for the imprisonment of host mRNA. The crystal structure has shown that ORF6 competitively binds to the mRNA-binding groove of the Rae1–Nup98 complex where the sidechain of a conserved methionine (M58) of ORF6 inserts into the hydrophobic pocket in Rae1, masking a large surface area. This results in the entrapment of RNAs inside the nucleus [137]. SARS-CoV-2 NSP1, on the other hand, targets other export receptors to achieve this goal. NXF1-NXT1, an mRNA export heterodimer, interacts with NPC to mediate the docking of mRNA on NPC [138]. NXF1-NXT1, an mRNA export heterodimer, interacts with NPC to mediate the docking of mRNA on NPC. SARS-CoV-2 NSP1 directly interacts with NXF1-NXT1 and wards off NXF1 docking on NPC and appropriate attachment of NXF1 with mRNA export adaptors, resulting in the detention of cellular mRNA in the nucleus [139].

### Global translation shutoff in the host cell

Although viruses depend on host translational machinery, they often use different tactics to impose selective control of the translational landscape in the host cells [140]. Among all HCoV proteins, NSP1 is the most well-characterized protein to impose the global shutoff of host translation. Structural analysis by cryo-EM revealed that the C-terminus of SARS-CoV-2 NSP1 interacts with the 40S ribosomal subunit, especially rRNA helix h18, and blocks the mRNA entry channel (Fig. 4) [141]. In another study, researchers noticed that the NSP1 C-terminus exhibits structural similarity with SERBP1 and Stm1, two known inhibitors of the ribosome that bind with the mRNA entry channel [142]. SARS-CoV NSP1 uses a two-pronged strategy to induce translational shutoff. It interacts with the 40S ribosomal subunit, forming a complex that modifies the 5’ region of the capped mRNA template and renders them translationally incompetent. Additionally, it induces RNA cleavage if templates carry internal ribosome entry sites (IRES) [143]. MERS-CoV NSP1 induces endonucleolytic cleavage of host RNA and prevents host protein production. R125 and K126 residues are crucial for this mRNA degradation function [144]. SARS-CoV-2 NSP1 also targets host mRNA for endonucleolytic cleavage-mediated degradation, thus facilitating the viral takeover of the mRNA pool and, hence, the selective translation of viral mRNA over cellular mRNA [145]. The first stem-loop (SL1) in the leader sequence is sufficient to prevent translation suppression. Thus, SL1 acts as a gatekeeper, and all SARS-CoV-2 RNAs that have viral leader sequences in the 5′ are safeguarded from this translation suppression [145, 146]. Apart from NSP1, SARS-CoV-2 NSP14 also has translational inhibition activity. Mutations in ExoN and N7-MTase active sites abolish their translation inhibition ability, suggesting that both activities are crucial. Detail mechanism is yet to be elucidated. However, NSP10 enhances this inhibitory activity [147].

After transcription, nascent pre-mRNA is processed by splicing to excise the introns and fuse the exons, forming mature RNA. It is conveyed by the spliceosome, a multiplex of noncoding RNA and proteins. Briefly, U1 small nuclear RNA (U1 snRNA) and U2 snRNA, two crucial components of the spliceosome, hybridize to the 5’ splice site at the exon-intron junction and branch point exons site, respectively, to initiate the splicing procedure [148]. In this context, SARS-CoV-2 infection induces nuclear translocation of NSP16, where NSP16 tethers to the splice site recognition sequence of U1 and the branchpoint recognition site of U2 and disrupting splicing of newly synthesized genes, resulting in a global increase of unspliced nascent RNAs (Fig. 4) [142]. During translation, a signal recognition particle (SRP) binds to the 80 S subunit of the translating ribosome and scrutinizes for hydrophobic sequences in the nascent peptide. Upon recognition, 80 S ribosomes are translocated to ER by SRP for proper protein folding and trafficking afterward [149]. NSP8 and NSP9 both are shown to interact with the S domain of the 7SL RNA scaffold of SRP. Here, NSP8 binds with 7SL in the SRP54 (responsible for signal peptide recognition) binding region, and NSP9 interacts with the SRP19 binding area (Fig. 4) [142].

### Proteolytic targeting of antiviral proteins

Viral proteases, involved in viral polyprotein processing, often cleave host proteins to suppress immune pathways (Fig. 2) [150]. SAR-CoV-2 proteases, PLpro and 3Clpro, have similar activities. Although SARS-CoV PLpro targets the ubiquitin chain, SARS-CoV-2 PLpro targets ubiquitin-like interferon-stimulated gene 15 protein (ISG15) from host proteins for cleavage [151]. SARS-CoV-2 NSP3 antagonizes the ISG-dependent activation of MDA5 by its deISGylating activity [152]. It also cleaves ISG15 from IRF3 and attenuates IFN response (deISGylation) [151]. MERS-CoV PLpro also has deISGylating and deubiquitinating activity, which reduces ISGylated and ubiquitinated host protein levels. Cysteine 1594 is the key residue for the catalytic activity [95]. A previous study on HCoV-NL63 shows the deubiquitinating and deISGylating role of NSP3 that blocks the IFN production [153]. Another study showed that SARS-CoV-2 NSP3 directly cleaves IRF3 at the LGGG sequence at residues 268–271 [154]. NSP5 of SARS-CoV-2, on the other hand, is shown to cleave NLRP12 [154]. 3Clpro targets NEMO, the essential modulator of NFκB, in brain endothelial cells [155]. NSP5 also cleaves off the ten most N-terminal amino acids from RIG-I, thus preventing its interaction with MAVS and downstream signaling (Fig. 2) [97]. The Oligoadenylate synthetase (OAS)-RNase L pathway gets activated upon recognition of viral dsRNA, and activated RNaseL cleaved viral and host ssRNA, resulting in translation inhibition and cell death, thus preventing virus spread. MERS-CoV ORF4b has phosphodiesterase (PDE) activity, enzymatically cleaves 2′,5′-oligoadenylate (2–5 A), activators of RNase L, therefore inhibits oligoadenylate synthetase (OAS)-RNase L pathway [156].

An overall summary of various strategies of HCoV proteins to evade or antagonize cellular innate immunity is summarized in Table 1.

**Table 1:** Human coronavirus proteins involved in IFN evasion and antagonism

Mechanism of inhibition	SARS	MERS	SARS-CoV2	Seasonal Coronaviruses
Inhibition of translation	**NSP1** (interacts with 40 S ribosomal unit and modifies 5’cap rendering incompetent for translation) [143]	**NSP1** (cleaves host mRNA) Orf4b (competitively binds to the nuclear import adapter IMPα3, thus inhibits NFkB-dependent innate immune pathway) [144]	**NSP1** (interact with 40 S ribosomal subunit, degrade mRNA) [141, 145, 146], **NSP9** (interacts with the S domain of 7SL and prevents its binding with SRP19) [142], **NSP14** [147], **NSP16** [142].	**NSP1** of **HCoV-NL63 and HCoV-229E** interact with and inhibit cellular translation machinery [157]
Inhibition of mRNA export	**ORF6** (competitively binds with the mRNA binding groove of Nup98-Rae1 nuclear export factor) [137]		**NSP1** (interacts with mRNA export heterodimer NXF1-NXT1 and prevents NXF1 docking on NPC and appropriate attachment of NXF1 with mRNA export adaptors) [139], **ORF6** (competitively binds to mRNA binding groove of Nup98-Rae1 and inhibits mRNA export from the nucleus) [137]	
Inhibits recognition of viral RNA- cap viral RNA	**NSP10**, **NSP13**, **NSP14** (N7-methyltransferase), **NSP16** (2’-O methyltransferase) [78]		**NSP10**, **NSP13**, **NSP14** (N7-methyltransferase), **NSP16** (2’-O methyltransferase) [79]	**NSP16** of **HCoV-229E** methyl transferase for the cap structure formation [62]
Block Viral RNA detection (DMV form)	**NSP3**, **NSP4**, **NSP6** [158]	**NSP3**, **NSP4** [159]	**NSP3**, **NSP4**, **NSP6**, **NSP15** (uridine-specific endonuclease activity cleaves the 5′-polyuridin sequence from negative-sense vRNA and prevents its accumulation) [75, 87, 90]	
Modifications of host proteins	**NSP3** (reduces the ubiquitinated levels of RIG-I, STING, TRAF3, TBK1, and IRF3 in the STING-TRAF3-TBK1 complex) [122]	**NSP3** (reduces the levels of ISGylated and ubiquitinated host protein) [95]	**NSP3** (deISGylation of MDA5 and IRF3) [151, 152]	**NSP3** of **HCoV-NL63**, **HCoV-HCU1**, **HCoV-229E**, and **HCoV-OC43** deISGylates of host proteins and Immune suppression [153, 160]
Cleave antiviral proteins			**NSP5** (cleaves the N-terminal amino acids of RIG-I, cleaves NLRP1, TAB, NEMO) [97, 154]	
Inhibiting activation of Innate Immunity proteins -RIG-I			**NSP5**, **ORF6** and **N** (prevents interaction of RIG-I and TRIM25, promote K48 ubiquitination) [91, 93, 94]	
Inhibiting activation of Innate Immunity proteins -MAVS	**ORF9b** (Alters mitochondria fission and disrupts MAVS/TRAF3/TRAF6 signaling) [161]		**NSP5** (degradation of MAVS) [97] **M** and** N** (Prevents MAVS aggregation formation and recruitment of downstream signaling components) [98, 99] **ORF9b** (Interacts with TOM70 and inhibits MAVS-dependent signaling) [101] **ORF10** Induces mitophagy-mediated degradation of MAVS by interacting with NiX and LC3B [100]	
Inhibiting activation of Innate Immunity proteins-STING	**NSP3** (disrupts STING mediated signaling) [123]		**NSP5** (inhibits K63 ubiquitination of STING) [124], NSP6 (autophagic degradation due to ER stress) [125], ORF3a (inhibits STING-mediated autophagic flux) [126]	**NSP3** of **HCoV-NL63** inhibits STING dimerization and further signaling [123]
TBK1	**M** protein prevents TRAF3·TANK·TBK1/IKKε complex formation [104]		**ORF9b**, **NSP13** (inhibits TBK1 phosphorylation) [107, 108], **M** (promotes degradation by K48-linked ubiquitination) [109]	
Inhibition of IRF3	**NSP3** (IRF3 dimerization) **N**, **ORF3b**, **ORF6**, **M** (prevents formation of TRAF3·TANK·TBK1/IKKε complex and thereby inhibits IRF3/IRF7 activation) [104]	**M** (inhibits nuclear translocation upon SeV infection, inhibits TRAF3-TBK1 association and IRF3 activation)[105], **ORF4a**, **ORF4b**, **ORF5** (IRF3 nuclear translocation) [112] **Orf4a** (prevents TBK1, IKK ε interaction with MAVS) and hence IRF3 activation [112]	**NSP5**, **NSP12**, **NSP13**, **ORF6** (nuclear translocation) **NSP6** (IRF3 phosphorylation), prevents IRF3 phosphorylation, and nuclear translocation by inhibiting IRF3-KPNA6 interaction [117–120]	
Inhibition of IFN signaling	**ORF6** (prevents STAT1 nuclear transport by sequestering import factors) [134]	**NSP3** (reduces production of proinflammatory cytokines like CCL5, CXCL10) [95], **M**, **ORF4a**, and **ORF4b** (reduces ISG54 and ISG56 mRNA level, thus ISRE promoter activation) [112]	**NSP13** (inhibits endogenous level of IFNAR) **NSP14** (Targets IFNAR for lysosomal degradation) [128] **NSP13** and **Spike** (STAT1 phosphorylation inhibition) [116, 129] **N** (inhibits interaction between STAT1/STAT2 and JAK1/TYK2 and reduces phosphorylation) [130] **ORF3a** (upregulates SOCS1, an E3 ligase that negatively regulates IFN signalling, and accelerates JAK2 degradation in ubiquitin-proteasome dependent manner) [131] **ORF7a** (inhibition of STAT2 phosphorylation) [135]	

## Human coronavirus mediated innate immune dysregulation

Coronaviruses are known to manifest a severe disease associated with cytokine storm, acute respiratory distress syndrome (ARDS), and, in critical cases, a multi-organ failure [162]. Higher mortality in SARS and MERS has been reported to be strongly correlated to leukopenia and lymphopenia, along with an exaggerated inflammatory response. Even though there are reports stating that human primary macrophages and macrophage-derived Dendritic cells (MDDCs) were found to be non-permissive to MERS infection [163], many studies report that, unlike SARS, MERS is capable of entering and replicating in Monocyte-derived Macrophages (MDMs) and even in T cells, besides the airway epithelium cells and produce cytokines and chemokines [164, 165]. A cytokine array study in 7 MERS patients reported an extremely high level of plasma type II IFN, IFN𝛾, and type I IFN, IFNα2. IFN𝛾 is secreted by CD4 T cells and NK cells, whereas IFNα2 is secreted by all types of cells, including the infected epithelium cells. TNFα, IL15, and IL17 levels were also reported to be high [166]. A comparative study of MERS and SARS infection in human-derived MDMs outlines the cytokine profile, which shows a stronger IFN𝛾 in MERS-infected MDMs compared to SARS infection [164]. Reportedly, a higher IL1B, IL12, IL6, and IFN γ is observed in SARS patients compared to higher IL15, IL17, TNFα, and IFN γ in MERS [167]. IL12 in SARS-CoV acts as an activation factor for CD8 + and NK cells, which again produce IFN γ. This positive feedback may result in abnormal cytokine levels and consequently lead to functional impairment or even apoptosis of T cells [168]. This trend of adverse immune response is also seen in SARS-CoV-2 disease COVID-19.

**Fig. 5:**
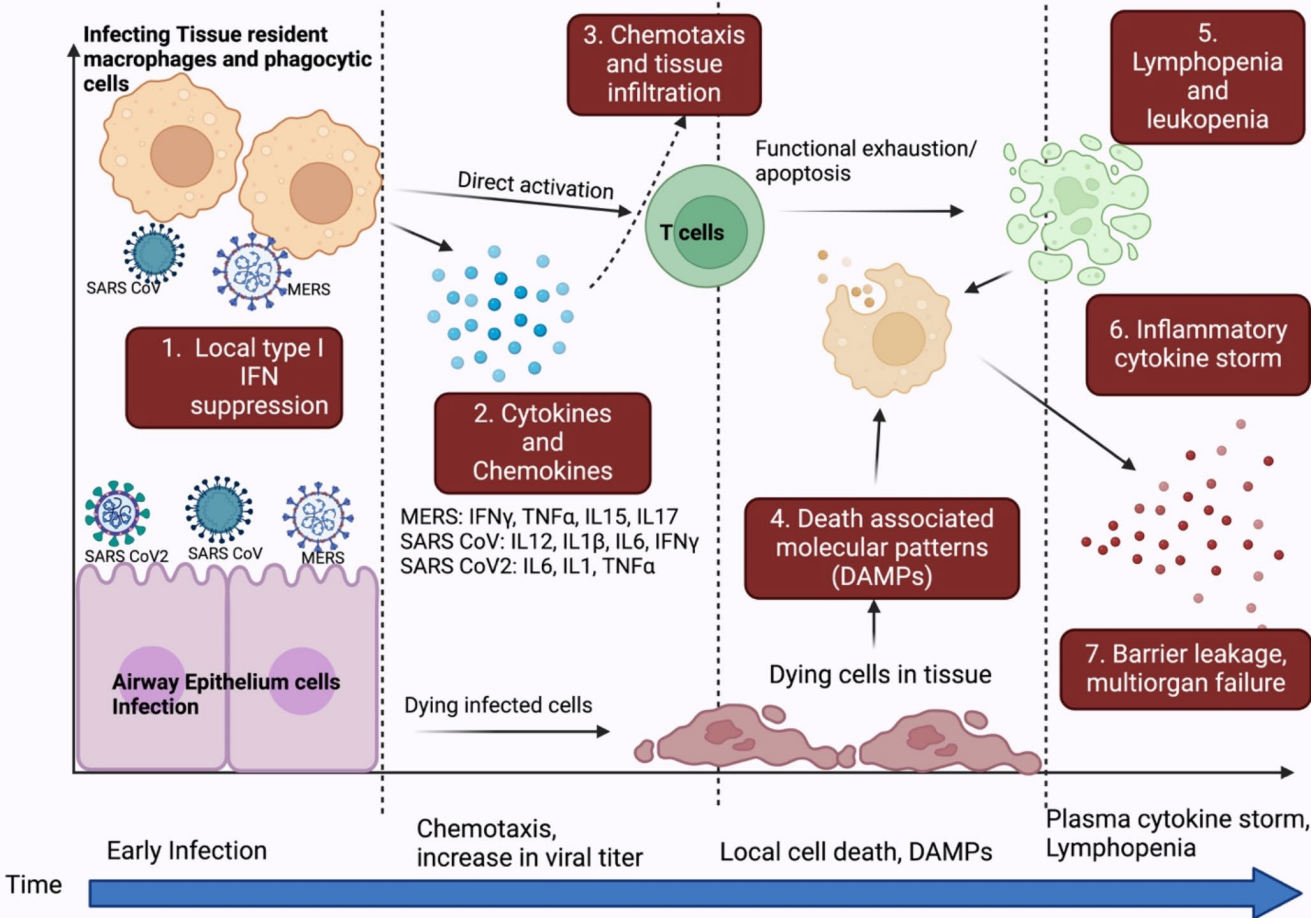
Dysregulation of IFN response by human coronaviruses. Coronaviruses employ an army of strategies to evade IFN induction and signaling at the cellular level, creating a replicative niche. In addition to airway epithelium, MERS and SARS infect macrophages and T cells. Local infection leads to the production of cytokines and chemokines that potentiate the chemotaxis of Lymphocytes and Neutrophils. In the later stages of the disease, local tissue damage may happen due to infection-related cell death, which may further enhance cytokine production and tissue infiltration. Lymphocytes are also subjected to functional exhaustion and death. In severe cases, there may be total respiratory collapse and cytokine storm

The majority of clinical studies have described COVID-19 symptoms taking a turn from a deficiency of sufficient IFN response initially to a hyperinflammatory response in the later stages of the disease. This temporally skewed IFN response may help the virus to establish a niche initially followed by enhanced dissemination (Fig. 5). Initial reports on the clinical relevance of IFN response to disease severity demonstrated a direct correlation between the severity of disease, essentially the hospitalization, with lower type I IFNs [169–171]. At the same time, hypercytokinemia, primarily high levels of IL6, was found to be positively associated with the severity of the disease. TLR2-dependent signaling induces the production of proinflammatory cytokines and directly correlates with disease severity [54]. Similarly, the TNFα pathway-dependent genes like IL-1 were also found to be highly expressed. Compared to mild COVID-19, severe COVID-19 patients had nearly 2.5 times lesser IFNα and 5-fold lesser IFNβ [171]. Another study reported functional impairment of the major innate immunity driving cells-plasmacytoid dendritic cells and plasma myeloid cells, wherein upon stimulation, the patient-derived cells led to lesser IFN and TNFα production. However, the plasma TNFα levels were high along with three more genes: TNFSF4 (ligand of B lymphotoxin receptor, inflammation-related gene), EN-RAGE (S100A12, pulmonary ARDS-related biomarker), and Oncostatin M (IL-6 regulator) [172]. IL-2, IL-7, TNF-α, Granulocyte colony-stimulating factor (G-CSF), IP-10, MCP-1, and MCP-1 A were reportedly higher in severe SARS CoV2 cases [168]. There is evidence of SARS-CoV2 infection in the immune cells-macrophages, and dendritic cells, which may be direct entry or phagocytosis of infected cells. However, this infection is abortive as per current evidence [173]. Similarly, patients with auto-antibodies to the type I IFN displayed greater disease severity [174]. Interestingly, in this study, nearly 10% of patients with life-threatening COVID-19 symptoms were found to have auto-immunity against type I IFN [174]. Contrary to these reports, another study reported an upregulation of IFN as well as other inflammatory cytokines, IL-6 and TNFα [175].

In the case of SARS-CoV-2, although the virus pushes for an insufficient innate immune response by inhibiting IFN production and signaling at various steps, STING-mediated inflammatory activity is upregulated. Various reports provide evidence for the SARS-CoV-2 spike-mediated cell-cell fusion that drives the cGAS-STING pathway [68]. These fusion events disturb nuclear compartmentalization, leading to micro-nucleus production and chromatin leakage into cytosolic compartments. Besides this, mitochondrial compartmentalization is also compromised, resulting in DNA fragments within the cytosol [70]. Essentially, these events lead to an aggravated STING activity overlapping with massive NFkB activity, resulting in TNFα production [68, 69, 176, 177]. Surprisingly, the ACE2 receptor is one of the ISGs and can potentially help enhance the infection [178]. Such immune dysregulation is also observed in SARS infection, wherein severe cases of infection also had pathological levels of IFN, ISGs, and other cytokines. A patient study in SARS-CoV also discovered a robust IFN response at an early stage of severe disease [179]. Another study in mice reported a delayed IFN response in SARS, which turned out to be lethal due to the high inflammatory cytokines secreted by monocytes and macrophages. Here, the popular belief of IFN as protective was challenged when IFNAR-/- mice had a milder disease than normal BALB/c [180].

In conclusion, severe COVID-19, MERS, and SARS symptoms can be ascribed to a suboptimal IFN response with confidence complemented by a hyperregulated cytokine response. However, the same cannot be stated for other respiratory coronaviruses since symptomatically coronavirus infection can range from mild common colds caused by HCoV-229E, -NL63, -OC43, and -HKU1 to moderate and moderate lethal for SARS CoV, SARS CoV2, and MERS [181]. Common cold-associated viruses HCoV-229E and NL63 were found to be more sensitive to IFN signaling-mediated inhibition compared to SARS-CoV2 and MERS [181]. Particularly in the case of moderate to lethal disease-causing coronaviruses, the viruses maneuver their way around the antiviral response locally very efficiently, causing local IFN deficit, such that the virus spreads and infects the macrophages and monocytes, generating an inflammatory state due to the cytokine storm and multi-organ failure in some cases. In contrast, the common cold-causing respiratory coronaviruses tend to maintain a replication niche in the upper respiratory tract and may be easily cleared off by the IFN response. However, there still exists a void in our understanding of the reason for deficit IFN production by the innate immune cells and a higher circulating plasma level of inflammatory cytokines. Some researchers have proposed this to be a sepsis-like condition stemming out of exposure of innate immunity cells and PRRs to Death-associated molecular patterns (DAMPs) and bacterial products (PAMPs) due to a breach of compartmentalization [182]. An in-depth and across–the-globe analysis of COVID-19 patient data is needed to establish a relationship between molecular factors of the host and the virus that drive the disease symptoms. Any medical intervention to curb disease symptoms needs to be based on a precise understanding of the patient’s IFN and cytokine load. Excessive inflammatory IFNs and cytokines like IL6 exacerbate COVID-19 pathology. This dysregulation of IFN response presents an opportunity to develop host-directed therapies targeting innate immune responses. One of the most prevalent therapies is IL-6 inhibitors like- Tocilizumab, an anti-IL6 antibody, and another antibody, Anakinra, which targets the IL-1 receptor. Such therapies have been reported to subdue the symptoms in critically ill patients, abating mortality [183, 184]. Other factors targeted include IL-17 (a master regulator of IL1 and IL6) and GM-CSF (a proinflammatory cytokine) [185]. Similarly, dysregulated IFN can be countered using IFN induction and signaling inhibitors, wherein critical patients can be given IFN agonists to fortify host antiviral defenses. Baricitinib (a JAK1 and JAK2 inhibitor) and Tofacitinib (JAK1, JAK2, and JAK3 inhibitor) reportedly benefitted hospitalized patients [74]. Thus, a carefully host-designed therapy can help counter the terminal COVID pathology, saving lives.

## Conclusion & perspective

The SARS-CoV-2 appeared in 2019 and remains the reason for one of the most significant global health burdens even in 2025. Comprehensive studies have revealed that many proteins of this virus have IFN antagonistic activity [118]; however, most have studied viral protein function in isolation and using *in vitro* systems. Thus, it is essential to validate these findings comprehensively using the reverse genetics approach and *in vitro* and *in vivo* models. Some IFN antagonists are uniquely encoded by SARS-CoV-2, which are not found in other *betacoronaviridae* members that infect humans. For example, SARS-CoV-2 shares adequate nucleotide sequence homology with SARS-CoV [186]; however, it has a unique accessory protein, ORF10 [187]. The function of this 38 amino acid accessory protein is yet to be elucidated, however some studies have shown that ORF10 overexpression inhibits the cGAS-STING pathway. It directly interacts with STING and impairs its association with TBK1, thus downregulating cGAS-STING-induced IRF3 activation. It also anchors STING in the ER, prevents translocation to Golgi, and antagonizes the innate antiviral pathway [127]. Another study showed that ORF10 deletion doesn’t change the replication ability and transmission of the virus [188, 189]. SARS-CoV-2 continues to change genetically through mutations and recombination. The structural proteins of SARS-CoV-2, especially spike and Not ucleoprotein, are the primary targets of adaptive immunity and continue to change to evade the same [190, 191]. Interestingly, the nonstructural and accessory proteins have also been evolving with each wave of COVID-19 and have altered the ability of the virus to evade innate immunity. In a dose titration experiment, the VOCs (alpha, beta, gamma, delta, and omicron) exhibited 5.1-fold and 4.4-fold higher IC_50_ for IFNβ and IFNλ1, respectively, compared to the ancestral lineage B isolates, thus showing increased resistance to IFNs [192]. Surprisingly, instead of increased adaption in suppressing specific host proteins, VOCs enhanced the expression of innate immune antagonist viral proteins [193]. The alpha variants showed upregulated sgRNA and protein levels of N, ORF6, and ORF9b, known inhibitors of the antiviral innate immune pathway [194]. While these genetic changes have made the virus more evasive and adept in IFN antagonism [195], it also gives a potential explanation for the reduced virulence potentially due to mitigated inflammatory response. These aspects need more comprehensive examination to understand viral evolution and its effect on virulence. Finally, it is now well established that SARS-CoV-2 infection can lead to a complex set of sequelae, collectively known as ‘Long COVID.’ The detailed molecular mechanisms and virus-host interactions leading to long COVID are not entirely understood; however, it will be interesting to examine the role of viral antagonists of innate immunity in the same, using reverse genetics and long COVID animal models [196].

Coronaviruses stand out among other RNA viruses’ for their ability to antagonize IFN-mediated antiviral response. Among HCoVs that caused epidemics, SARS-CoV-2 shares 79% sequence homology with SARS-CoV and 50% sequence homology with MERS at the genetic level [197]. The case fatality rate (CFR) varies among these viruses, with MERS being the most fatal with 30–40% CFR and SARS-CoV-2 being the least fatal with 1% CFR [4]. Dissecting the molecular basis of this variation in the severity of the disease is critical to establish a regimen for treatment. More than half of the viral proteins of these coronaviruses contribute to immune evasion and dysregulation. These viral proteins likely act in a complementary manner to inhibit different innate immune pathways concomitantly to achieve the maximum pro-viral state. For example, ORF3a has the unique ability to inhibit STING but not the RLR pathway. On the other hand, the N protein inhibits the RLR pathway without affecting STING, suggesting viral proteins act cooperatively [124]. Thus, the systemic deletion of different viral proteins can illuminate such a cooperative effect and pave the way for potential attenuated vaccine candidates to fight the virus. In this review, we have focussed on the IFN pathway and its modulation by HCoVs. However, other cellular processes such as autophagy, ER-Stress, and cell death pathways also create an innate antiviral response [198–200]. This needs more detailed examination, especially for defining differential viral replication and pathogenesis among HCoVs. Compared to humans, one understudied aspect of HCoVs is their immune evasion mechanisms in the wild reservoir hosts, especially in Bats. Bats are known to have a subdued innate immune response to viral pathogens [201]. Likely, coronaviruses that emerged from bats (SARS, MERS, and potentially SARS-CoV-2) may have acquired mutations in viral proteins that make them differentially susceptible or resistant to the human innate immune system. Examining this aspect of HCoV can shed light on factors contributing to viral emergence from wild reservoirs that lead to human infection and its spread. To conclude, human coronaviruses are a clinically significant group of viral pathogens. They have already contributed to a few major outbreaks and a global pandemic and are likely to cause more in the future [202]. Considerable work has been done to understand the key HCoV viral proteins that modulate cellular innate immunity. However, much remains unexplored, especially in the context of viral evolution and pathogenesis. Continued comprehensive research on these aspects of HCoVs will enhance our preparedness for future pandemics.

## Data Availability

No primary data was generated.
